# Transmembrane carbonic anhydrase isozymes IX and XII in the female mouse reproductive organs

**DOI:** 10.1186/1477-7827-2-73

**Published:** 2004-10-17

**Authors:** Piritta Hynninen, Jonna M Hämäläinen, Silvia Pastorekova, Jaromir Pastorek, Abdul Waheed, William S Sly, Eija Tomas, Pertti Kirkinen, Seppo Parkkila

**Affiliations:** 1 Department of Gynecology and Obstetrics, University of Tampere and Tampere University Hospital, Tampere, Finland; 2 Institute of Medical Technology, University of Tampere and Tampere University Hospital, Tampere, Finland; 3 Center of Molecular Medicine, Institute of Virology, Slovak Academy of Sciences, Bratislava, Slovak Republic; 4 Edward A. Doisy Department of Biochemistry and Molecular Biology, Saint Louis University School of Medicine, St. Louis, Missouri, USA

## Abstract

**Background:**

Carbonic anhydrase (CA) classically catalyses the reversible hydration of dissolved CO_2 _to form bicarbonate ions and protons. The twelve active CA isozymes are thought to regulate a variety of cellular functions including several processes in the reproductive systems.

**Methods:**

The present study was designed to investigate the expression of transmembrane CAs, CA IX and XII, in the mouse uterus, ovary and placenta. The expression of CA IX and XII was examined by immunoperoxidase staining method and western blotting. CA II and XIII served as positive controls since they are known to be present in the mouse reproductive tract.

**Results:**

The data of our study indicated that CA XII is expressed in the mouse endometrium. Only very faint signal was observed in the corpus luteum of the ovary and the placenta remained mainly negative. CA IX showed weak reaction in the endometrial epithelium, while it was completely absent in the ovary and placenta.

**Conclusion:**

The conservation of CA XII expression in both mouse and human endometrium suggests a role for this isozyme in reproductive physiology.

## Background

Carbonic anhydrases (CAs) are zinc-containing metalloenzymes that are responsible for the reversible hydration of carbon dioxide in a reaction CO_2 _+ H_2_O ↔ H^+ ^+ HCO_3_^-^. CAs are produced in a variety of tissues where they participate in several important biological processes such as acid-base balance, respiration, carbon dioxide and ion transport, bone resorption, ureagenesis, gluconeogenesis, lipogenesis and body fluid generation [1,2]. The mammalian α-CA gene family includes at least twelve enzymatically active isoforms with different structural and catalytic properties. CA I, II, III, VII and XIII are cytosolic enzymes [1,3,4]. CA VA and VB are mitochondrial proteins encoded by nuclear DNA [5,6]. CA VI is the only secretory form being present in saliva and milk [7]. The cluster of membrane-bound CAs includes four isozymes: CA IV, IX, XII, and XIV [8-11]. The other members of the CA gene family (CA VIII, X and XI) are inactive isoforms whose functions have not yet been described [3,12,13].

It has been previously suggested that CAs may play important roles in the uterine endometrium by maintaining the appropriate pH balance through the catalysis of the production of bicarbonate ions [14]. Indeed, the role of bicarbonate in fertilization has been demonstrated in a number of previous studies. It is functionally involved in some key processes such as sperm cell capacitation and regulation of sperm motility [15-17]. Similarly, CAs may have several functions also in the placenta. They can be active in intermediary metabolism and provide ions for exchange in transepithelial movement of ions and fluid [18].

CA activity has been studied in pig, horse, cow, mink, rat and human placentas, and the results show considerable heterogeneity among different species [18]. Previous immunochemical studies have shown evidence for expression of CA II but not CA I or III in the bovine placenta [19]. Both CA I and II are expressed in the human syncytiotrophoblasts [20-22] and, especially CA II, in the fetal villous endothelium of mature placenta [22]. CA IV-positive staining has been reported in the mouse placenta by Rosen and coauthors [23]. Their data showed strong CA IV immunoreactivity in the mouse trophoblasts and endodermal layer of the yolk sac. In the mouse genital tract, CA I, II and III have been reported by Ge and Spicer [24]. These isozymes were reported to be present in the theca interna cells in the mouse ovary, and CA I was found in the zona pellucida and cytoplasmic foci in follicular granulosa cells. In the mouse oviductal epithelium, CA II expression showed distinct variation. The reaction was absent in the infundibulum, whereas the ampulla and isthmus showed positive staining. CA XIII is the newest member of the CA enzyme family, which has been described in the mouse and human endometrium along with several other positive tissues [4]. As a cytosolic isozyme it may be one of the major proteins regulating the pH and bicarbonate homeostasis not only in the endometrial cells but also in the lumen of the uterus. These mechanisms are complex due to the presence of several isozymes, however, and may greatly differ between species. For example, the human endometrium contains CA II only in the capillaries, whereas this high activity isozyme is abundantly expressed in the epithelial cells of the mouse endometrium [4,24].

CA IX is expressed at the basolateral plasma membrane of the human, rat and mouse epithelial cells [25,26]. In a recent extensive study, Ivanov *et al *[27] analyzed a number of normal human tissues for the expression of CA IX. Among reproductive organs, they reported positive signal for CA IX mRNA and protein in the efferent ducts, rete testis, and rete ovarii.

Human CA XII is expressed in several organs including colon, kidney, and pancreas [28-30]. In the human female reproductive tract, CA XII has been shown both in the glandular and surface epithelium of the endometrium, while it was only occasionally present in the cervix [14]. Ivanov *et al *[27] further confirmed CA XII expression in the glandular epithelium during the proliferative phase.

In this report we studied the expression of CA II, IX, XII and XIII in mouse female genital organs including uterus, ovary and placenta. The studies were specially focused on CA IX and XII, which have been designated as tumor-associated isozymes [9,10]. In addition to some normal tissues, both isozymes are overexpressed in several carcinomas such as renal and colorectal cancers [9,27,31,32]. A previous study has also demonstrated CA IX and XII expression in a number of neoplasias derived from the female reproductive tract [27]. However, there have been no previous studies on these isozymes in the female murine reproductive organs. The conservation of CA XII expression in both mouse and human endometrium shown in the present paper suggests a role for this isozyme in reproductive physiology.

## Materials and methods

### Antibodies

In the present study, we used the following antibodies which have been produced and characterized earlier: rabbit anti-mouse CA II [4], rabbit anti-mouse CA IX [26], rabbit anti-mouse CA XII [33], and rabbit anti-mouse CA XIII [4].

### Collection of tissue samples

Two adult Balb/c mice were sacrificed by CO_2 _asphyxiation followed by decapitation. Uterus, ovary and placenta samples were collected from both animals. The samples were immersion-fixed overnight in Carnoy's fluid (ethanol, chloroform and acetic acid (6:3:1)). Then the specimens were treated with absolute ethanol for 30 min, with 1:1 mixture of ethanol and chloroform for 15 min, and finally with chloroform for 30 min. Paraffin embedding was performed in a vacuum oven for 2 h at +58°C. Paraffin wax was purchased from Fluka Chemie GmbH (Buchs, Schwitzerland). To collect a placenta sample, a mouse was sacrificed at 9 days of pregnancy. The ninth day was chosen since it represents the middle gestational phase. It is also the time when the most critical steps of organogenesis occur in mouse. For western blotting, uterus, kidney and colon were removed and rapidly frozen in liquid nitrogen. The tissue samples for western blot were homogenized with HEPES buffer. Total protein concentration was determined after homogenization using BCA Protein Assay Kit (Pierce, Rockford, IL) according to manufacturer's instructions. The study protocols were approved by the Animal Care Committee of Tampere University.

### Immunohistochemistry

In the mouse tissues, the localization of CA IX and XII was examined by immunoperoxidase method. Antibodies against CA II and XIII were used as positive controls for the immunostaining. All experiments were performed in duplicate and included control staining with non-immune normal rabbit serum (NRS). NRS was obtained from a rabbit that was later immunized against mouse CA XIII. The tissue samples fixed in Carnoy's fluid and embedded in paraffin were cut at 5 μm sections and placed on microscope slides. The peroxidase-anti-peroxidase complex method included the following steps: a) pretreatment of the sections with undiluted cow colostral whey (Biotop, Oulu, Finland) for 40 min and rinsing in phosphate-buffered saline (PBS); b) incubation for 1 h with the primary antiserum (anti-mouse CA II, CA IX, CA XII or CA XIII) or NRS diluted 1:100 in PBS containing 1% bovine serum albumin (BSA) (BSA-PBS solution); c) treatment with undiluted cow colostral whey (40 min); d) incubation for 1 h with swine anti-rabbit IgG (Dakopatts, Copenhagen, Denmark) diluted 1:100 in 1% BSA-PBS; e) incubation for 30 min with peroxidase-anti-peroxidase rabbit conjugate (Dakopatts) diluted 1:500 in PBS; f) incubation for 2 min with 3,3'diaminobenzidine tetrahydrochloride (DAB) solution (6 mg DAB in 10 ml PBS plus 3.3 μl H_2_O_2_) as chromogen. The sections were washed three times for 10 min in PBS after incubation steps b and d and four times for 5 min after incubation step e. All of the incubations and washings were carried out at room temperature. The sections were finally mounted in Neo-Mount (Merck, Darmstadt, Germany). The stained sections were examined and photographed with a Zeiss Axioskop 40 microscope (Carl Zeiss, Göttingen, Germany).

### Western blot

The samples containing 50 μg of protein from mouse uterus, kidney and colon were analyzed by SDS-PAGE under reducing conditions [34]. All of the reagents and the protein standard (BenchMark™ Prestained Protein Ladder) for SDS-PAGE were purchased from Invitrogen (Carlsbad, CA) except Laemmli sample buffer that was obtained from Sigma (St. Louis, MO). Electrophoresis (200 V for 40 min) was performed in a Novex Xcell II mini cell electrophoresis unit (Invitrogen) with a 10% Bis-Tris gel (Invitrogen). The separated proteins were transferred electrophoretically from the gel to a polyvinylidene fluoride (PVDF) membrane (Invitrogen) in a Novex Xcell II blot module (Invitrogen). The transfer buffer (NuPAGE Transfer Buffer™) was purchased from Invitrogen. The blot module was filled with the transfer buffer until the gel/membrane assembly was covered. The outer buffer chamber was filled with 650 ml deionized water. The protein transfer was performed using a constant voltage of 36 V for 1 h 20 min. After the transblotting, the sample lines were detected by ECL western blotting detection reagents and analysis system (Amersham Biosciences, Buckinghamshire, UK) according to the manufacturer's instructions. First, the sample lines were incubated with TBST buffer (10 mM Tris-HCl, pH 7.5, 150 mM NaCl, 0,3 % Tween 20) containing 10 % cow colostral whey for 25 min and then the first antibodies diluted 1:2000 (anti-CA II, anti-CA IX, anti-CA XII, NRS) or 1:1000 (anti-CA XIII) in TBST buffer for 1 h. The PVDF membranes were washed five times for 5 min with TBST buffer and incubated for 1 h with peroxidase-linked ECL Anti-Rabbit IgG (Amersham Biosciences) diluted 1:25 000 in TBST buffer. After washing four times 5 min in TBST buffer, the polypeptides were visualized by a chemiluminescence substrate (ECL detection reagents 1 + 2, Amersham Biosciences). Kodak™ Biomax™ MS-1 films (Amersham Biosciences) were exposed to the chemiluminescence for 5 min (CA IX and XII) or 1 min (CA II and CA XIII). All the steps were carried out at room temperature. The western blotting experiments were performed in triplicate to confirm the reproducibility of the results.

## Results

### Immunohistochemistry

**Figure 1:**
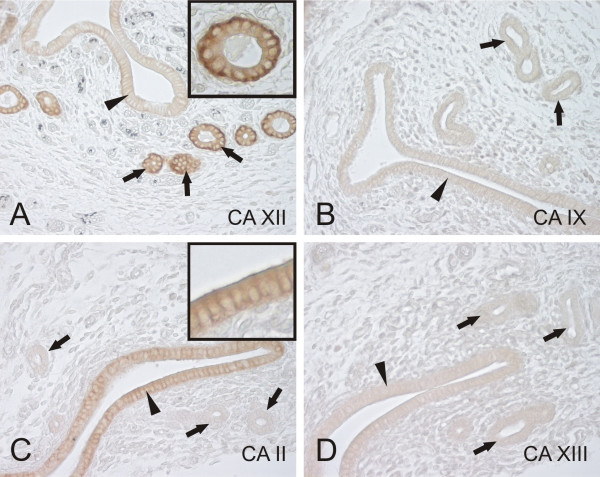
Immunohistochemical staining of CA XII (A), CA IX (B), CA II (C), and CA XIII (D) in the mouse endometrium. All the studied CA isozymes show positive immuostaining, although the staining intensity varies between different isozymes. CA XII shows stronger reaction in the deep endometrial glands compared to the surface epithelium. This pattern is inversed with CA II showing high reaction in the surface epithelium. Insert in panel A shows that the CA XII immunostaining is most abundant in the basolateral plasma membrane of the epithelial cells. Insert in panel C demonstrates that CA II immunoreactivity is also closely associated with the plasma membrane. CA IX and XIII show faint immunoreactions in both the surface and glandular epithelia. Arrows = endometrial glands, arrowheads = surface epithelium. Original magnifications: × 400.

**Figure 4:**
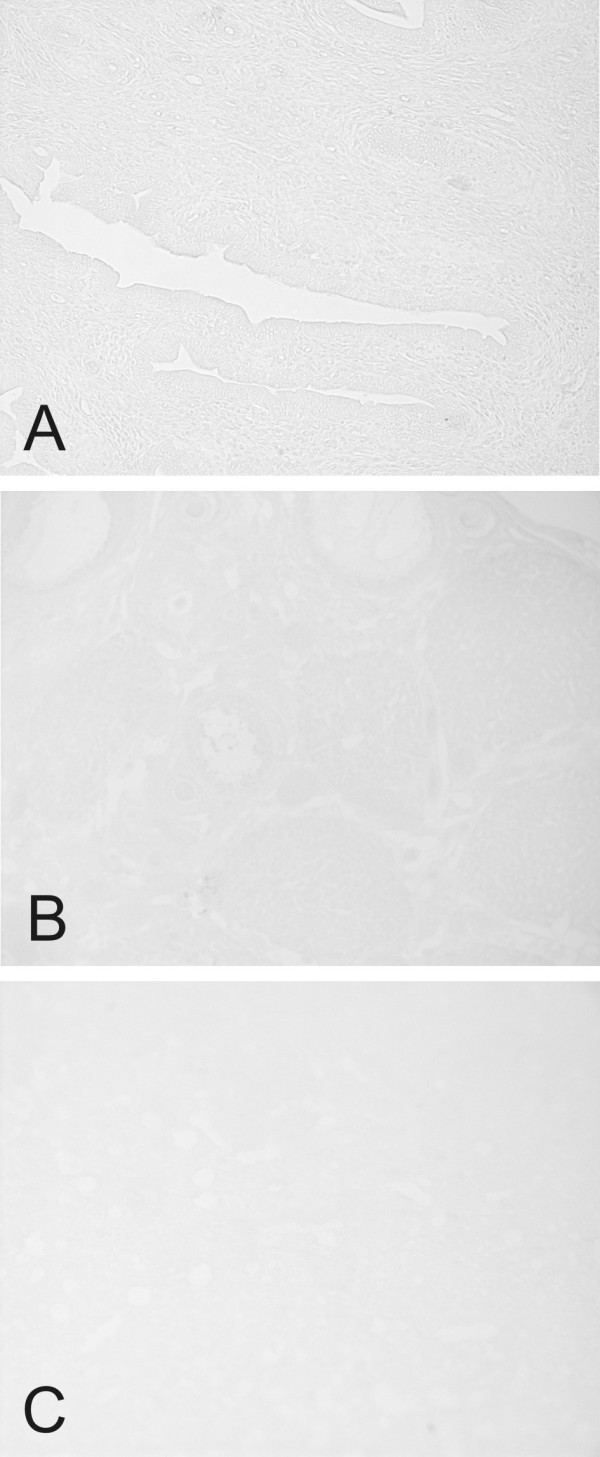
Control immunostaining of mouse uterus, ovary and placenta with normal rabbit serum. No immunoreaction is seen. Oringinal magnifications: × 400.

All the studied CA isozymes showed positive immunostaining in the epithelial cells of the mouse endometrium (Fig. 1). CA II and XII showed a somewhat reciprocal distribution pattern in that CA II was confined to the surface epithelial cells (Fig. 1C), while CA XII was more intensely stained in the deeper endometrial glands (Fig. 1A). It is noteworthy, however, that CA XII was clearly expressed also in the surface epithelial cells, but the staining intensity was weaker compared to the glands. As expected, the strongest reaction for CA XII was associated with the basolateral plasma membrane, and unexpectedly, also CA II immunoreaction was most intense at the plasma membrane. CA IX and XIII showed weak reactions in both surface and glandular epithelia (Fig. 1B,1D). The control immunostaining with NRS was negative (Fig. 4A).

**Figure 2:**
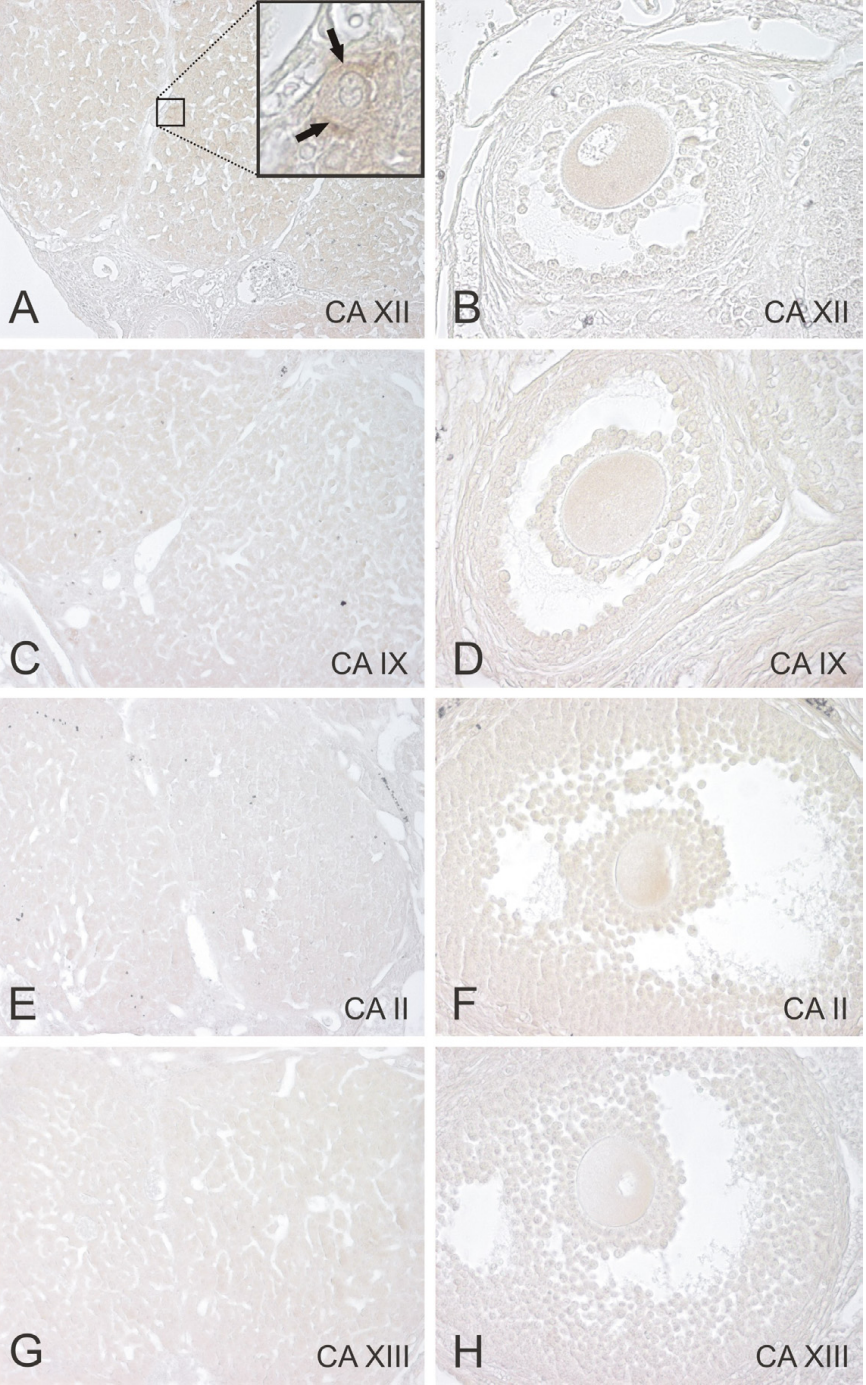
Immunolocalization of CA XII (A,B), CA IX (C,D), CA II (E,F), and CA XIII (G,H) in the mouse corpus luteum (A,C,E,G) and follicle (B,D,F,H). Only faint positive reaction for CA XII can be seen in occasional cells of the corpus luteum that is indicated in the insert of the panel A (arrows). Original magnifications: × 200 (A,C,E,G), × 400 (B,D,F,H).

In the ovary, immunoreactions for different CA isozymes were negligible (Fig. 2). In fact, only CA XII showed occasional positive cells in the corpus luteum (Fig. 2A). No staining for these isozymes was observed in the developing follicles. No immunoreaction was obtained with NRS (Fig. 4B)

**Figure 3:**
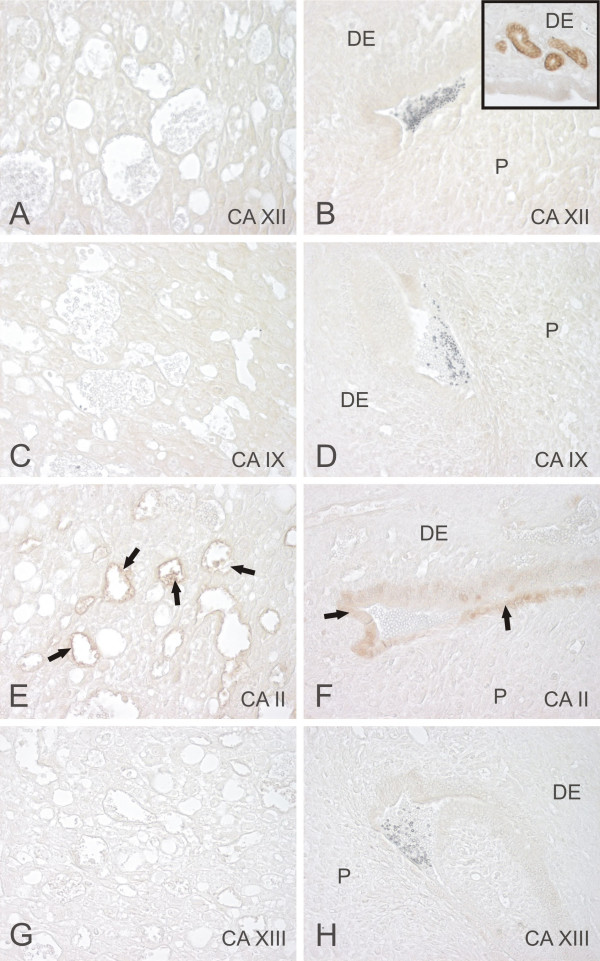
Immunohistochemical staining of CA XII (A,B), CA IX (C,D), CA II (E,F), and CA XIII (G,H) in the mouse placenta (A,C,E,G) and amnionic epithelium (B,D,F,H). CA II is located in the endothelium of the blood vessels and erythrocytes (arrows in the panel E). It is also expressed in the amnionic epithelium (arrows in the panel F). Insert of the panel B shows that CA XII is highly expressed in the decidual glands, while the amnionic epithelium is negative. DE = Decidua, P = placenta. Original magnifications: × 400.

In the 9-days-old mouse placenta, the immunostaining reactions for CA isozymes remained quite weak or absent (Fig. 3). CA II was located to the endothelium of the placenta blood vessels and erythrocytes (Fig. 3E), and it was also present in the amnionic epithelium (Fig. 3F). The amnionic epithelium showed no or very weak staining for CA XII, whereas the decidual glands were strongly labeled (Fig. 3B). The control staining again showed no positive signal (Fig. 4C).

### Western blot

**Figure 5:**
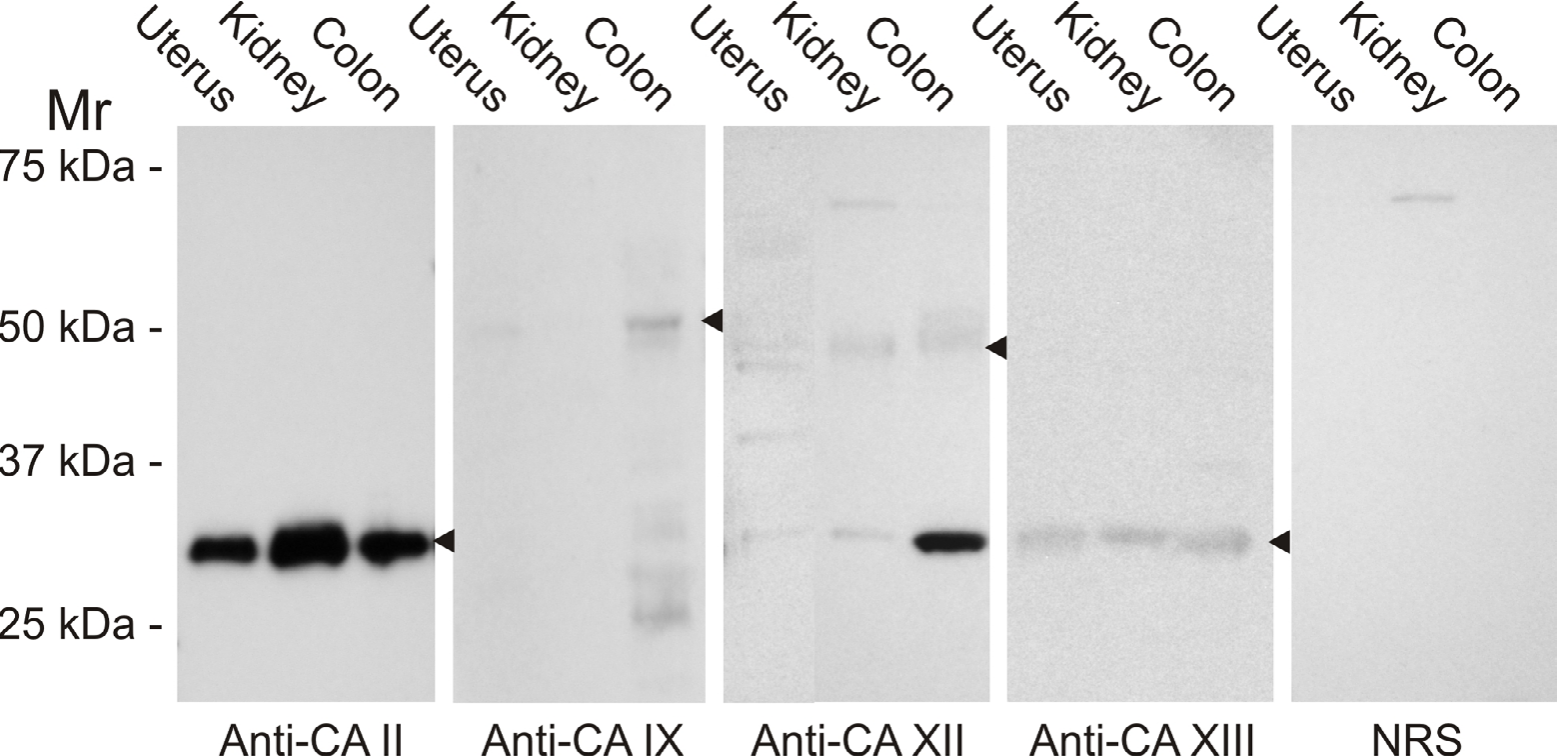
Western blotting of total homogenate from mouse uterus, kidney and colon for CA II, IX, XII and XIII. Normal non-immune rabbit serum (NRS) was used instead of the first antibodies as a negative control. Both CA IX and XII show weak positive reactions for the uterine proteins (arrowheads). The molecular weights for these isozymes are 51 and 46 kDa, respectively. The signal for CA XII is weaker in the uterus than in the colon or kidney. Note that anti-CA XII serum cross-reacts with a 30-kDa polypeptide. This cross-reaction is evident only in western blotting conditions as pointed out in the Results section. CA IX shows the strongest signal in the colon. CA II and XIII are positive in all tissue specimens.

Western blotting was performed for the mouse uterine protein to evaluate the specificity of the immunoreactions. Mouse kidney and colon samples were used as positive control tissues, since they are known to express CA II, XII and XIII [4,33,35], and the colon contains CA IX [36]. CA II and XIII were positive in all tissue specimens (Fig. 5). Both CA IX and XII showed weak positive reactions in the mouse uterus. The molecular weights for these isozymes were 51 and 46 kDa, respectively. Based on the western blotting the expression of CA XII was weaker in the uterus than in the colon or kidney. On the other hand, CA IX showed the strongest signal in the colon. It is notable that anti-mouse CA XII serum cross-reacted with 30 kDa polypeptide in the western blotting. Previous immunostaining of gastric mucosa with the same anti-CA XII and anti-CA II antibodies has clearly indicated that anti-CA XII serum does not recognize CA II which has a molecular mass of 30 kDa in western blot [35]. Even though gastric epithelial cells contain high levels of CA II, no immunoreaction was obtained with anti-CA XII serum in those cells. Furthermore, no staining has been obtained by anti-CA XII antibody in the red cells which contain high levels of CA I and II, nor in the brain which expresses high levels of CA II and XIII (data not shown).

## Discussion

This study describes the expression of CA II, IX, XII and XIII in mouse female genital organs including uterus, ovary and placenta. CA II showed a very limited distribution pattern in the mouse placenta, being present only in the erythrocytes, endothelium of the blood vessels and amnionic epithelium. In previous studies, CA II has been detected by immunohistochemistry in the human villous syncytiotrophoblasts and in varying amounts in fetal villous endothelium [21,22]. Using a histochemical staining method, Ridderstråle et al [18] showed in several species that the highest CA activity located in the maternal capillaries, and the membrane-bound CA activity varied among different species. To date, CA IV is the only membrane-bound CA isozyme which has been detected in the mouse placenta [23]. In our study, CA IX and XII were not found in the mouse placental tissue except that CA XII showed a very weak reaction in the amnionic epithelium. Concluding from the results of the present and previous studies, CA I and II appear to represent the enzyme forms that are most relevant for the placental function [22], while CA IX and XII may play a role in other reproductive organs such as the male excurrent duct and female uterus [14,37].

It is known that CA activity facilitates transport of CO_2 _across biological membranes by converting it to bicarbonate and hydrogen ions. These ions are then translocated across the plasma membrane through specific carrier proteins in a coordinated manner. It is of considerable interest that CA isozymes II and IV have been recently described to form active metabolon systems with ion exchanger proteins such as anion exhanger isoform 1 (AE1) and Na^+^/H^+^-exhanger isoform 1 (NHE1) [38-40]. Even though these associations have not yet been described in the placental tissue, it is possible that such metabolons play a role in facilitating placental ion transport processes.

Previous studies have shown CA activity in the endometrium of several mammalian species [41,42]. Until now the only established isozymes in the human endometrium are CA XII [14] and CA XIII [4]. Interestingly, the high activity isoenzyme, CA II, is not expressed in the human endometrial epithelium [4]. In the present study, all the examined CA isozymes – including CA II – showed positive immunostaining in the epithelial cells of the mouse endometrium. To our knowledge, there are only a few examples of clear species-specific difference in CA expression. These include e.g. CA XII expression in the kidney (human principal cells versus mouse intercalated cells) [33,43] and CA XIII in the human and mouse testis [4]. What would be the physiological consequence of such variation between different species? Of course, there are marked differences in human and rodent reproductive physiology. Mouse is characterized by tremendous reproductive potential. Females generally have 5–10 litters per year if conditions are suitable. Gestation period is 19–21 days. Litters consist of 3–12 (generally 5 or 6) offspring, and the mice reach sexual maturity at 5–7 weeks. Even though our observations do not provide any clues whether CA expression could contribute to some of the described characteristics, these differences may have fundamental physiological effects that should be addressed in future investigations.

In the present study, CA XII and II showed more intense staining in the surface endometrial epithelia than CA IX and XIII. CA XII was more intensely stained in the deeper endometrial glands, while CA II was confined to the surface epithelial cells. Interestingly, CA II showed positive immunoreaction not only in the cytoplasmic compartment but also at the plasma membrane of the cells that is quite surprising for a cytosolic enzyme. The same phenomenon is detectable in some other tissues including the human gallbladder [25] and gut [44]. The cell membrane reactivity may reflect a possible physical association between CA II and ion transport proteins, which has been demonstrated in cell cultures [38-40]. When CA XII was first discovered in the normal human endometrium, it was suggested to play a role in reproductive functions [14]. In the endometrium, pH and ion balance has to be tightly regulated to ensure normal fertilization. For example, the bicarbonate concentration has been implicated in the regulation of sperm motility, capacitation, and acrosome reaction [15,17,45]. One major regulatory pathway includes a bicarbonate-sensitive adenylate cyclase that is present in the plasma membrane of the sperm cell [46]. In the female genital tract, the endometrial and oviductal epithelium may produce an alkaline – bicarbonate rich – environment for maintaining the sperm motility. This suggestion is in agreement with the observations by Guerin et al. [47], that the sperm motility is improved by co-culture of human spermatozoa with either endometrial or oviductal epithelial cells.

In the future studies, it will be important to investigate whether the hormonal status regulates the expression of different CA isozymes – particularly CA XII – in the endometrium. Another interesting line of investigations would be to analyze the fertilization capacity of CA XII knockout mice as soon as they become available. One could hypothesize that endometrial CA isozymes are important factors, contributing to the appropriate bicarbonate concentration and pH balance in the cervical and endometrial mucus needed for normal fertilization process. Based on our recent studies, CA IX-deficient mice showed no apparent phenotypic changes linked to fertility [26]. Even more interesting from this point of view is that CA XII may be an important isozyme present in the endometrium, and therefore, CA XII knockout mice will be very attractive targets for reproductive physiological studies.

## Conclusions

The present paper demonstrates for the first time the expression of transmembrane carbonic anhydrase isozymes IX and XII in the female murine reproductive tract. The data indicates that the endometrial epithelium is a prominent site for CA XII expression. The conservation of CA XII expression in the endometrium of different species (mouse and human) suggests a role for this isozyme in reproductive physiology.

## Authors' contributions

All authors participated in the design of the study. PH, JL and SP collected the tissue samples. PH, JL, ET, PK and SP drafted the manuscript. PH, JL and SP performed the western blotting. SPas, JP, AW and WSS provided the antibodies. PH, JL and SP participated in the immunohistochemical staining. All authors read and approved the final manuscript.
